# Supplementary material for: An Optimized Method to Culture Human Primary Lung Tumor Cell Spheroids
Source: Cancers (Basel). 2023 Nov 25;15(23):5576. doi: 10.3390/cancers15235576 (PMC10705303; doi:10.3390/cancers15235576)
Supplement: Supplementary file 1 [file cancers-15-05576-s001.zip › Mueggler A - Supplementary Figure S1.pdf]

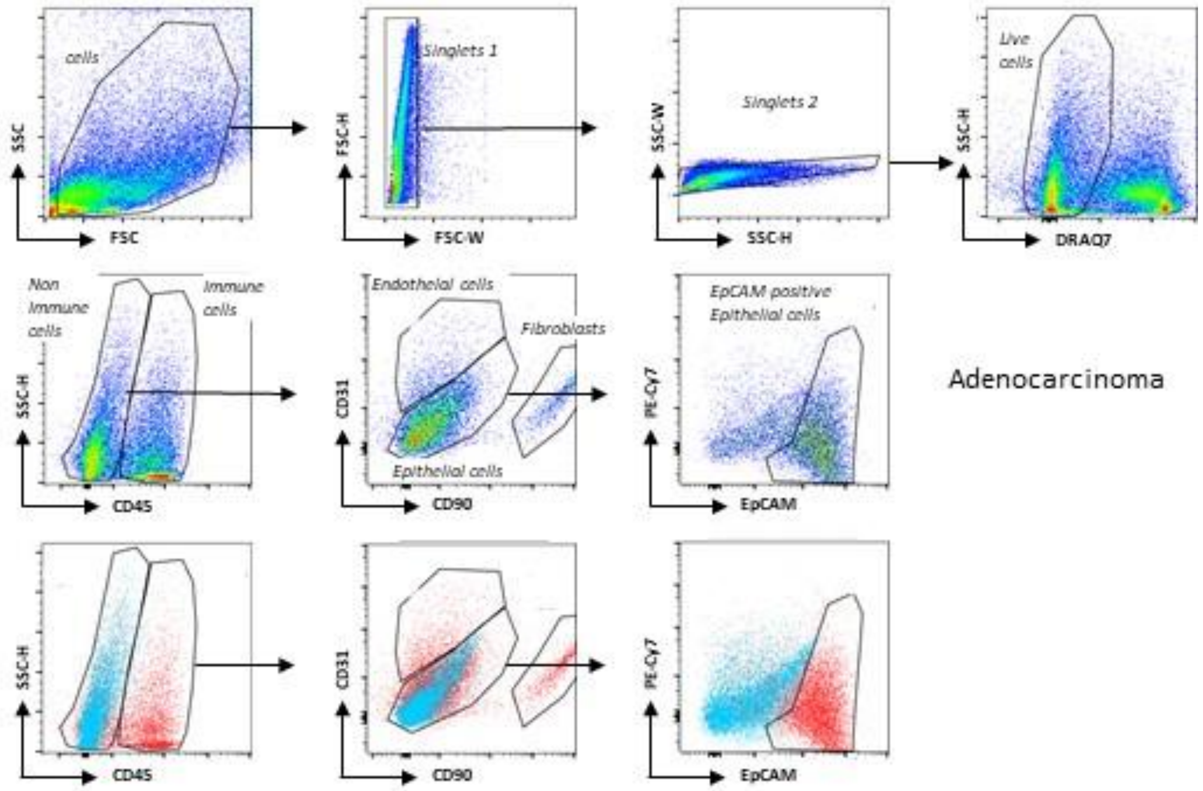

Figure S1. Gating strategy for flow cytometry analysis of human normal lung tissue and NSCLC samples. Gating strategies used for identifying cell subpopulations including immune cells and non-immune cells (fibroblasts, endothelial cells and epithelial cells) present in patient samples. Cells were gated on singlets 1 and 2 and gated out for dead cells using DRAQ7. Cells were then gated on CD45neg non-immune cells and analyzed for CD90 (Fibroblasts), and CD31 (endothelial cells) expression. In non-immune cells, epithelial cells were selected as CD90NegCD31Neg cells. The expression of the epithelial marker, EpCAM, was determined in this epithelial subpopulation.
