# Supplementary material for: An Optimized Method to Culture Human Primary Lung Tumor Cell Spheroids
Source: Cancers (Basel). 2023 Nov 25;15(23):5576. doi: 10.3390/cancers15235576 (PMC10705303; doi:10.3390/cancers15235576)
Supplement: Supplementary file 1 [file cancers-15-05576-s001.zip › Mueggler A - Supplementary Figure S2.pdf]

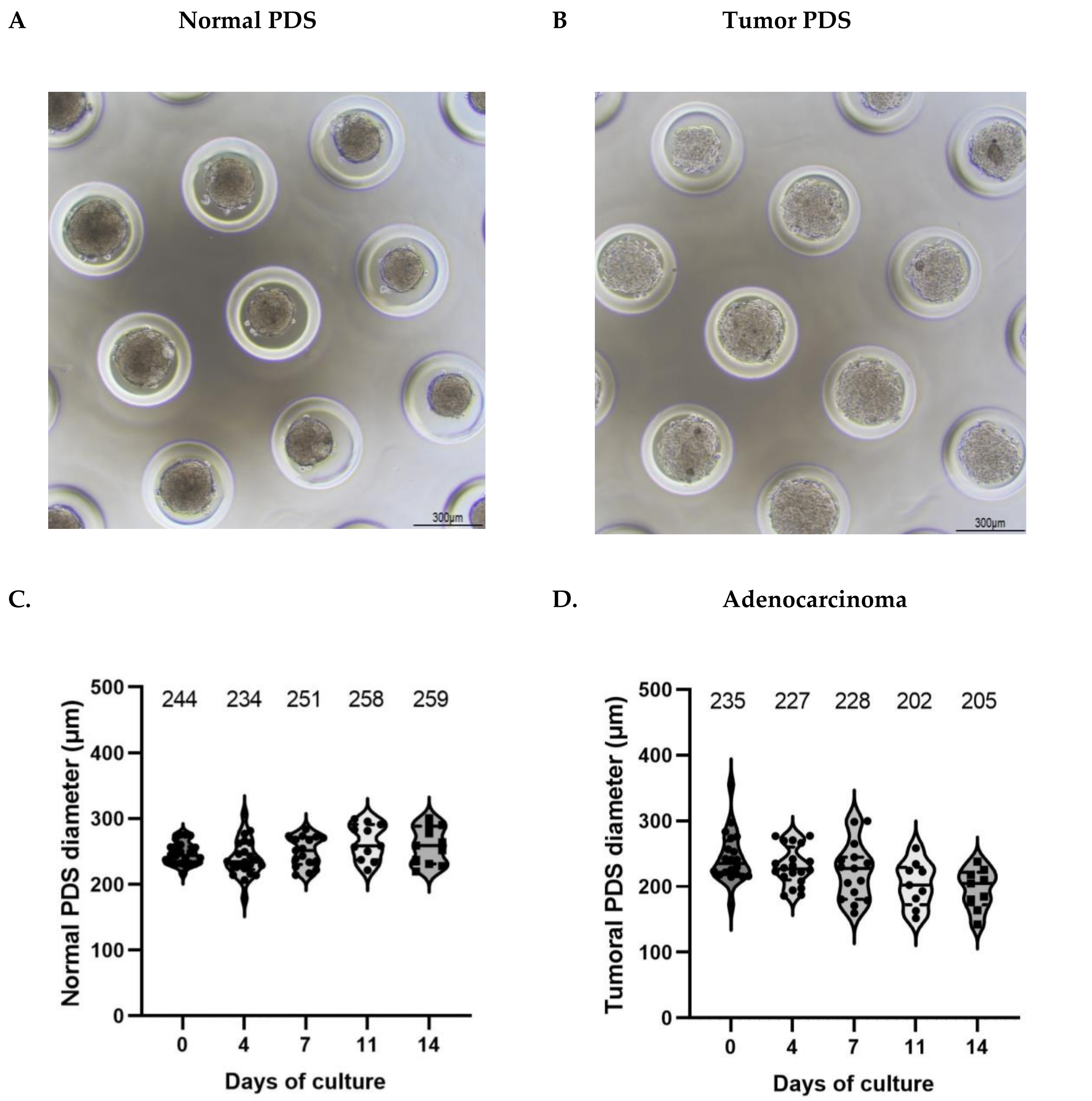

Figure S2. Growth of PDS cultured in PnExP medium. Representative images of Normal (**A**) and tumor (**B**) PDS established from the lung tissues of a patient diagnosed with a lung SqCC after 7 days of culture in PnExP medium. Violin plots of normal PDS (**C**) and adenocarcinoma PDS (**D**) diameter over 14 days of culture. The data represent the median ± quartiles of 15 to 20 spheroids.
