# Supplementary material for: An Optimized Method to Culture Human Primary Lung Tumor Cell Spheroids
Source: Cancers (Basel). 2023 Nov 25;15(23):5576. doi: 10.3390/cancers15235576 (PMC10705303; doi:10.3390/cancers15235576)
Supplement: Supplementary file 1 [file cancers-15-05576-s001.zip › Mueggler A - Supplementary Figure S3.pdf]

Lung adenocarcinoma – 19LuCa04

Carboplatin / pemetrexed

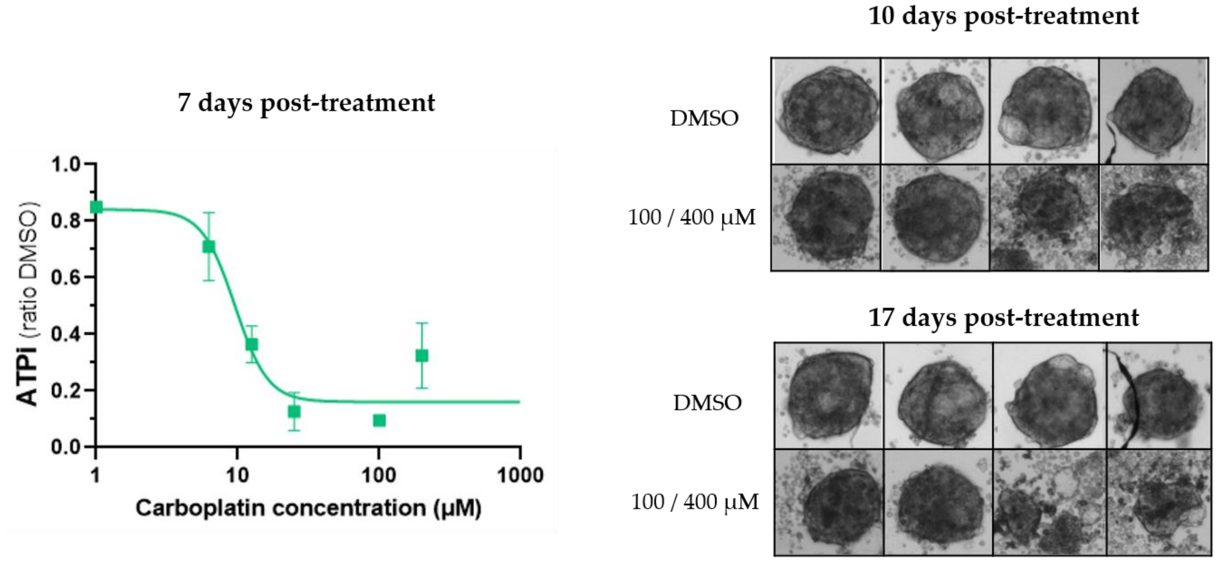

Figure S3. Dose-dependent effects of carboplatin/pemetrexed on lung adenocarcinoma PDS. The effect of the treatment was determined by assessment of PDS viability (left panel) at day 7 after treatment using intracellular ATP (ATPi) and PDS morphology (right panels) at day 10 and 17 after treatment using microscopy.
