# Supplementary material for: An Optimized Method to Culture Human Primary Lung Tumor Cell Spheroids
Source: Cancers (Basel). 2023 Nov 25;15(23):5576. doi: 10.3390/cancers15235576 (PMC10705303; doi:10.3390/cancers15235576)
Supplement: Supplementary file 1 [file cancers-15-05576-s001.zip › Supplementary file S1.pdf]

**Table S1.** Key Resources Table

| Reagent/Resource | Label | Identifier |
|---|---|---|
| Chemical, enzymes, and other reagents | | |
| Advanced DMEM/F12 | PAN™Biotech | P04-41300 |
| Hepes | GIBCO | 15630-056 |
| Glutamax | GIBCO | 35050 |
| Collagenase type I | PAN™Biotech | PANLS0004194 |
| Collagenase type II | PAN™Biotech | PANLS0004174 |
| Collagenase IV | Thermo Fisher Scientific | 17104019 |
| Elastase | Alfa Aesar | ALFJ61753-MB |
| DNAse I | Roche | 10104159001 |
| Red blood cell lysis buffer | Biolegend | 420301 |
| Penicillin/Streptomycin | PAN™Biotech | P06-07100 |
| TrypLE Express | GIBCO | 12605-010 |
| HBSS | GIBCO | 14170-088 |
| PBS | GIBCO | 10010-015 |
| Noggin | Peprotech | 120-10C |
| A83-01 | Cayman | 9001799 |
| Y-27632 | Cayman | 10005583 |
| B27 supplement | GIBCO | 12587-010 |
| Human bFGF | R&Dsystems | 234-FSE-025 |
| Human EGF | R&Dsystems | 236-EG-200 |
| Pneumacult™-Ex medium | Stemcell Technologies | 05008 |
| Pneumacult™-Ex Plus medium | Stemcell Technologies | 05040 |
| Hydrocortisone | Stemcell Technologies | 07925 |
| Mounting medium | BioSystems | 401603391 |
| EDTA | Applichem | A1104, 0500 |
| Bovine serum albumin (BSA) | Applichem | A1391, 0500 |
| Agarose | Sigma | A9539-100G |
| Antibodies and reagents for flow cytometry and immunofluorescence | | |
| Cytokeratin 7 | Dako | M7018 |
| Cytokeratin 5/6 | Chemicon | MAB1620 |
| p63 protein | Novocastra | NCL-L-p63 |
| Thyroid Transcription Factor (TTF1) | Dako | M3575 |
| Anti-mouse Alexa Fluor™ 488 | ThermoFisher | A-11001 |
| Anti-mouse Alexa Fluor™ 555 | ThermoFisher | A-21422 |
| ProTaqs Mount Fluor Anti fading medium | Biosystems | 401603392 |
| BV605 anti-CD326 (EpCAM) | BD | 563182 |
| PE/Cy7 anti-human CD56 (NCAM) | Biolegend | 362510 |
| APC anti-human CD45 | Biolegend | 304012 |
| BB700 anti-human CD31 | BD | 566564 |
| PE CD90 | BD | 555596 |
| BV605 mouse IgG1κ | Biolegend | 566404 |
| PE mouse IgG1κ | Biolegend | 400111 |
| PE/Cy7 mouse IgG1κ | Biolegend | 400125 |
| BB700 mouse IgG1κ | BD | 566404 |
| DRAQ7 | Biolegend | 424001 |
| Human TruStain FcX™ | Biolegend | 422302 |
| Others | | |

| | | |
|---|---|---|
| ATPlite™ 1stSTEP | PerkinElmer AG | |
| InviTrap Spin Universal RNA mini kit | Invitek Molecular | 1060100200 |
| Random Hexamer | GIBCO | 48190-011 |
| Reverse transcriptase Superscript II | Invitrogen | 18064-014 |
| dNTPs 10 mM | Life Technologies | 10297-018 |
| Takyon™ No Rox SYBR Master Mix dTTP Blue | Eurogentec | UF-NSMT-B0701 |
| MicroTissues® 3D Petri Dish® micro-mold spheroids | Thermofisher | Z764000 |
| Cisplatin | Teva Pharmaceutical Industries | |
| Etoposide | Selleck Chemicals | S1225 |
| Paclitaxel | Selleck Chemicals | S1150 |
| CELLSTAR®, Cell-Repellent Surface 96 Well-microplate | Greiner Bio-One | 7.650 0 |

**Table S2.** Antibodies used for the immunofluorescence staining.

| Antibodies | Manufacturer | Ref | Dilution |
|---|---|---|---|
| Cytokeratin 7 | Dako | M7018 | 1/50 |
| Cytokeratin 5/6 | Chemicon | MAB1620 | 1/50 |
| p63 protein | Novocastra | NCL-L-p63 | 1/100 |
| Thyroid Transcription Factor (TTF1) | Dako | M3575 | 1/100 |
| Ki67 | Cell signaling | 9027 | 1/500 |
| Anti-mouse Alexa Fluor™ 488 | ThermoFisher | A-11001 | 1/500 |
| Anti-mouse Alexa Fluor™ 555 | ThermoFisher | A-21422 | 1/500 |

**Table S3.** The sequences of gene-specific primers for RT-qPCR.

| Gene | Froward | Reverse |
|---|---|---|
| CADM1 | 5′-AGCCTTGGCCTCTTTCCTCAG-3′ | 5′-TCCCGAGGCTCCCCATCTAAG-3′ |
| KRT16 | 5′-TTGCTGGATGTGAAGACGCGG-3′ | 5′-GTGAAGACCTCGCGGGAAGAA-3′ |
| MMP1 | 5′-ATGGGCTTGAACTGCTTACGA-3′ | 5′- ACATTCTGTCCCTGAACAGCC-3′ |
| NAPSA | 5′-TATGGAACTGGGCGGGTAGA-3′ | 5′-GGGAAAACCGAGGCCCAATA-3′ |
| RPLP0 | 5′-GAAGACAGGGCGACCTGGAAG-3′ | 5′-GCGCATCATGGTGTTCTTGCC-3′ |
| HPRT1 | 5′-ACAGGACTGAACGTCTTGCTCG-3′ | 5′-TGATGTAATCCAGCAGGTCAGCA-3′ |
| GusB | 5′-ACGTGGTTGGAGAGCTCATT-3′ | 5′- CTCTGCCGAGTGAAGATCCC-3′ |
