# Supplementary material for: An Optimized Method to Culture Human Primary Lung Tumor Cell Spheroids
Source: Cancers (Basel). 2023 Nov 25;15(23):5576. doi: 10.3390/cancers15235576 (PMC10705303; doi:10.3390/cancers15235576)
Supplement: Supplementary file 1 [file cancers-15-05576-s001.zip › Supplementary file S2.pdf]

Table S4. Patient's clinical characteristics. Clinical characteristics of patients whose resected lung tissues were used for cell isolation and culture. Adenocarcinoma (ADC), Squamous cell carcinoma (SqCC).

Patient ID	Sex	Age	Tumor histotype	Tumor Stage
19LuCa01	F	77	SqCC	T3 pN0 R0
19LuCa02	F	60	ADC	T2 N0 R0
19LuCa03	M	75	Adenoid	T4 pN0 R0
19LuCa04	M	50	ADC	T1c N0 R0
19LuCa05	F	79	ADC	T1b N0 R0
19LuCa06	M	65	ADC	T1b pN1 R0
19LuCa07	F	75	SqCC	T2b pN0 R0
19LuCa08	F	67	ADC	T3 pN0 cM0
19LuCa09	M	77	ADC	T2a N0 R0
19LuCa10	M	74	ADC	T4 pN0 R0
19LuCa11	M	76	ADC	T1a N0 R0
19LuCa12	M	63	ADC	T2b N0 R0
19LuCa13	M	62	ADC	T3 N2 R0
19LuCa14	F	75	ADC	T1b N0 Rx
19LuCa15	F	57	SqCC	T2a N0 R0
19LuCa17	F	69	SqCC	T1c N0 pR0
19LuCa18	M	53	SqCC	T4 N0
19LuCa19	F	68	ADC	T2a N0 R0
19LuCa20	M	78	ADC	T4 N0 R0
19LuCa21	M	86	ADC	T2a N2 M1a
20LuCa02	F	61	ADC	T1c N1 R0
20LuCa03	M	67	SqCC	T1c N0 R0
21LuCa01	M	75	SqCC	T4 N0 R0
21LuCa02	M	65	ADC	T1c N0 R0
21LuCa03	F	58	SqCC	T3 N0 R0
21LuCa04	M	69	Carcinoid	T2b N2 R1
21LuCa05	M	58	SqCC	T1c N1 R0
21LuCa06	M	57	ADC	T3 N1 R0
21LuCa08	M	52	ADC	T4 N0 R1
21LuCa09	M	71	SqCC	T2b N0 R0

Table S5. Cell subpopulations in the digested lung tissues. Proportion of the different cell subpopulations obtained after the enzymatic digestion of the human lung normal and tumor tissues analyzed by multiparameter flow cytometry. Stat.: Wilcoxon matched-pairs signed rank test.

Patient ID	histotype	Viable cells DRAQ7^{Neg}	Hematopoietic cells CD45^{Pos}	Endothelial cells CD31^{Pos}	Fibroblasts CD90^{Pos}	Epithelial cells CD31^{Neg} CD90^{Neg} EpCAM^{Neg}	Epithelial cells CD31^{Neg} CD90^{Neg} EpCAM^{Pos}
			% of viable cells	% of CD45^{Neg}	% of CD45^{Neg}	% of CD45^{Neg}	% of CD45^{Neg}
**Normal lung (N)**							
21LuCa02		83.4%	91.0%	18.0%	29.0%	60.0%	8.8%
21LuCa03		85.6%	70.0%	7.0%	4.0%	81.8%	13.0%
21LuCa04		92.8%	68.3%	15.7%	31.7%	35.2%	16.1%
21LuCa05		89.1%	72.2%	34.6%	4.4%	24.1%	33.3%
21LuCa06		95.5%	40.0%	2.0%	51.0%	42.0%	0.0%
21LuCa08		91.1%	76.3%	11.0%	2.1%	54.0%	32.9%
21LuCa09		88.2%	83.5%	13.9%	13.0%	62.5%	6.8%
**Median**		**89.1%**	**72.2%**	**13.9%**	**13.0%**	**54.0%**	**13.0%**
**CI**		85.6–92.8	68.3–83.5	7.0 - 18.0	4.0–31.7	35.2–62.5	6.8 –32.9
**Tumoral lung (T)**							
19LuCa02	ADC	42.6%	59.9%	2.2%	6.0%	29.0%	64.5%
19LuCa03	Adenoid	75.7%	63.9%	5.0%	15.0%	40.0%	41.0%
19LuCa04	ADC	97.0%	30.4%	1.5%	2.8%	50.2%	44.8%
19LuCa05	ADC	90.4%	64.3%	2.3%	12.3%	14.4%	71.5%
19LuCa06	ADC	91.0%	37.6%	0.0%	3.3%	37.8%	59.5%
19LuCa07	SqCC	96.5%	3.5%	0.0%	0.4%	76.6%	19.0%
19LuCa08	ADC	89.5%	91.0%	2.2%	3.0%	54.4%	41.0%
19LuCa09	ADC	84.2%	59.6%	1.7%	9.0%	75.0%	13.0%
19LuCa12	ADC	88.6%	70.5%	2.5%	12.0%	70.0%	18.0%
19LuCa13	ADC	78.8%	73.4%	5.0%	12.0%	57.0%	27.0%
19LuCa14	ADC	80.5%	77.7%	3.0%	13.0%	36.8%	48.0%
19LuCa15	SqCC	83.2%	12.0%	0.0%	0.5%	42.6%	58.0%
19LuCa17	SqCC	74.7%	45.6%	0.6%	3.6%	85.9%	8.1%
19LuCa20	ADC	50.5%	39.0%	48.2%	43.7%	6.0%	0.0%
21LuCa02	ADC	90.3%	68.0%	6.0%	8.8%	42.3%	47.0%
21LuCa03	SqCC	92.0%	51.3%	1.0%	23.0%	74.0%	0.0%
21LuCa04	Carcinoid	95.5%	21.8%	3.2%	93.0%	5.1%	0.0%
21LuCa05	SqCC	95.5%	15.0%	1.7%	71.3%	24.0%	2.5%
21LuCa06	ADC	97.0%	31.6%	0.9%	79.0%	15.4%	4.3%
21LuCa08	ADC	93.1%	78.9%	2.0%	13.0%	78.9%	3.7%
21LuCa09	SqCC	94.9%	81.7%	3.5%	6.8%	77.9%	11.3%
**Median**		**90.3%**	**59.6%**	**2.2%**	**12.0%**	**42.6%**	**19.0%**
**CI**		79.7–95.2	31.0–72.0	1.0-3.4	3.5–19.0	26.5–74.5	4.0–47.5
**N vs T p value**		0.0313	0.1094	0.0313	0.3125	0.4688	0.4688

Table S6. Cell subpopulations in the cells from normal and tumor lung tissues after monolayer culture with the PnExP medium. Proportion of the different cell subpopulations obtained after culture in monolayer with the PnExP medium of the cells obtained from digested human lung normal and tumoral tissues analyzed by multiparameter flow cytometry. Stat.: Wilcoxon matched-pairs signed rank test.

Patient ID	histotype	Viable cells DRAQ7^{Neg}	Hematopoietic cells CD45^{Pos}	Endothelial cells CD31^{Pos}	Fibroblasts CD90^{Pos}	Epithelial cells CD31^{Neg} CD90^{Neg} EpCAM^{Neg}	CD31^{Neg} CD90^{Neg} EpCAM^{Pos}
			% of viable cells	% of CD45^{Neg}	% of CD45^{Neg}	% of CD45^{Neg}	% of CD45^{Neg}
**Normal lung (N)**							
19LuCa03		85.7%	1.0%	0.8%	0.4%	13.6%	83.6%
19LuCa06		79.9%	0.4%	1.2%	0.7%	40.3%	23.3%
21LuCa01		86.7%	0.0%	0.3%	0.0%	5.3%	93.1%
21LuCa02		88.0%	1.3%	0.0%	0.3%	11.0%	81.8%
21LuCa03		89.8%	0.0%	1.7%	0.6%	6.7%	91.5%
21LuCa04		93.1%	1.4%	0.5%	2.1%	19.5%	74.6%
21LuCa05		91.2%	0.5%	0.4%	0.3%	9.5%	89.5%
21LuCa08		91.5%	0.9%	0.4%	0.3%	14.0%	85.9%
**Median**		**88.9**	**0.7**	**0.5**	**0.4**	**12.3**	**84.8**
**CI**		86.0−91.4	0.1−1.2	0.3−1.1	0.3−0.7	7.4−18.1	76.4−91
**Tumoral lung (T)**							
19LuCa03	Adenoid	87.1 %	0.23 %	0.25 %	1.46 %	25,57%	71.9 %
19LuCa06	ADC	61.4%	0.0%	2.2%	6.5%	21.3%	66.8%
19LuCa20	ADC	83.2%	0.8%	1.9%	0.8%	30.8%	66.5%
20LuCa03	SqCC	81.0%	27.9%	9.1%	11.0%	22.7%	48.0%
21LuCa01	SqCC	81.6%	1.4%	6.7%	24.5%	28.8%	29.8%
21LuCa02	ADC	84.8%	1.8%	3.4%	0.1%	12.0%	84.0%
21LuCa03	SqCC	86.4%	0.8%	0.7%	3.5%	14.4%	80.3%
21LuCa04	Carcinoid	77.8%	14.2%	1.7%	92.8%	2.8%	1.6%
21LuCa05	SqCC	91.0%	0.1%	0.0%	0.3%	59.1%	36.9%
21LuCa06	ADC	90.1%	2.8%	0.0%	1.1%	7.2%	91.0%
21LuCa08	ADC	90.7%	2.0%	0.4%	1.5%	31.8%	66.4%
**Median**		**84.0**	**1.6**	**1.8**	**2.5**	**22.0**	**66.5**
**CI**		80.2−90.3	0.6−5.7	0.3−4.2	0.4−14.4	10.8−31.1	35.1−81.2
**N vs T p value**		0.0391	0.1406	0.2344	0.0313	0.3125	0.1094
